# Factors, Perceptions and Beliefs Associated with Inappropriate Antibiotic Prescribing in German Primary Dental Care: A Qualitative Study

**DOI:** 10.3390/antibiotics10080987

**Published:** 2021-08-16

**Authors:** Femke Böhmer, Anne Hornung, Ulrike Burmeister, Anna Köchling, Attila Altiner, Hermann Lang, Christin Löffler

**Affiliations:** 1 Institute of General Practice, Rostock University Medical Center, 18057 Rostock, Germany; altiner@med.uni-rostock.de (A.A.); christin.loeffler@med.uni-rostock.de (C.L.); 2 Rostock University Library, Rostock University Medical Center, 18059 Rostock, Germany; anne.hornung@med.uni-rostock.de; 3 Department of Operative Dentistry and Periodontology, Rostock University Medical Center, 18057 Rostock, Germany; ulrike.burmeister@med.uni-rostock.de (U.B.); hermann.lang@med.uni-rostock.de (H.L.); 4 Clinic for Psychosomatic Medicine and Psychotherapy, Rostock University Medical Center, 18147 Rostock, Germany; anna.koechling@med.uni-rostock.de

**Keywords:** qualitative research, antimicrobial resistance, dentistry, inappropriate prescribing, anti-bacterial agents

## Abstract

Dentists account for up to 10% of all prescribed antibiotics in primary care, with up to 80% being inappropriate. Targeted approaches to change prescription behavior are scarce. This study aimed at identifying specific barriers and facilitators for prudent antibiotic use in German dentistry by using qualitative methods. Nine in-depth interviews and two focus group discussions with another nine dentists were conducted and analyzed thematically. Dentists described being conflicted by the discordance of available treatment time and the necessity of thorough therapy. Lacking the opportunity of follow-up led to uncertainty. Dentists felt a lack of medical competency concerning prophylaxis for infectious endocarditis. A lack of empowerment to make therapeutic decisions interfered with guideline-conformity. The communication with fellow physicians is conflictual and improvement was wished for. In consequence, dentists felt pressure by potential medico-legal liability. Patients demanding quick and easy pain relief put extra strain on the interviewed dentists. Our hypotheses concord with preliminary data, mainly from the UK, but highlighted specifically medico-legal concerns and interprofessional communication as even greater barriers as described before. Tailored interventional concepts based on our findings may have the potential to lower antibiotic prescriptions in German primary dental care.

## 1. Introduction

Antimicrobial resistance through overuse and misuse of antibiotics is one of the most urgent problems in worldwide health care. The link to the extent of antimicrobial consumption has undeniably been established. Globally, a rise in the consumption of critically important antibiotics by 91% has been noted between the years 2000 and 2015 and this trend has not halted, especially in low- and middle-income countries [1]. Despite the pressing need for action and decreasing trends in some countries, including Germany, recent data do not show any significant reduction in overall outpatient antibiotic use in Europe. Moreover, a continuous trend towards the use of broad spectrum antibiotics can be observed [2].

General dental practitioners (GDP) account for up to 10% of all antibiotic prescriptions in European outpatient care [3,4]. Within this specialty a non-guideline conformity towards both indication of antibiotic prescribing and the choice of the antimicrobial substance has been implied [4,5,6,7]. Studies have shown that up to 80% of antibiotic prescriptions might be inappropriate in acute dental conditions as local operative procedures are predominantly sufficient [8]. Dental pathogens have already shown alarming levels of resistance [9,10]. Moreover, increased dental antibiotic prescribing has been triggered by the SARS-CoV-2-pandemic, as recent data from England has shown [11].

As of now, there are few data on the prescriptions of antibiotics in German primary dental care. These side with pre-known international evaluations and show a prescribing proportion of 8.8% of all outpatient disciplines. Remarkably, clindamycin remains one of the most prescribed antibiotics in German dentistry [12,13,14]. This is worrying as clindamycin is associated with high numbers of adverse events compared to amoxicillin [15].

Considering the above, empowering antibiotic stewardship in dentistry becomes a key issue in implementation research. Intervention studies aiming at decreasing antibiotic use in primary care have shown positive effects [16], however, so far there are only few studies intending to modify dental behavior. Of those, most do not fulfil standards for interventional studies, but they yield some encouraging results [17,18,19,20,21,22,23,24,25,26,27,28,29].

To facilitate behavior changes in health care professionals towards guideline-conformed antibiotic prescribing, interventions need to be as targeted as possible. Barriers and facilitators should be precisely addressed. Yet, only few qualitative studies aiming at understanding dentists’ views towards antibiotics have been conducted [30,31,32,33,34]. Most insights on possible confounders to irrational prescribing have been acquired within Anglo-American settings, predominately in the UK. To our knowledge, so far there are no studies on this matter in the specific setting of German dentistry [14,30,35].

Our study thus aimed at thoroughly exploring dentists’ experiences, attitudes and perceptions related to antibiotic treatment in German dentistry and gaining a deeper understanding on reasons for non-indicated antibiotic prescribing using a qualitative approach.

## 2. Results

The qualitative data revealed multi-layered aspects linked to non-rational antibiotic prescribing. The following themes were extracted to be of greater significance. Sample descriptions of the interviews and focus group discussions are presented in Table 1.

### 2.1. Lack of Time

All interviewees perceived themselves as “low- prescribers”. Asked to quantify this, reported prescriptions rates reached from five a month to twice a year. An exception was the maxillofacial surgeon interviewed, who prescribed for every third patient undergoing surgical procedures.

Nonetheless, the majority stressed that they would be more inclined to prescribe antibiotics in particular situations. Subsumed under “lack of time”, these included patients with acute dental pain consulting without appointments either during emergency medical services or immediately before the start of the patients’ holidays or before weekends. Dentists explained that they worried about the potentially worsening health condition of patients. Moreover, any lack of possibility to follow-up the patients’ further health situations or to change the therapy regimen if needed contributed to the dentists’ insecurities. Consequently, dentists were in favor of prescribing an antibiotic as perceived safety measurements:

*“In acute cases, where I would actually need to treat the patient, but he shows up in the emergency service and I accordingly don’t have time or possibilities to sufficiently treat the patient, I tend to shield with an antibiotic. Though, thank god, that doesn’t happen very often. But in those situations […] I do doubt, whether it was excessive therapy, whether it was really appropriate.” (male dentist, 29 years, urban)*

Some shortness of time to adequately treat their patients was described. Cases of failed endodontic treatment served as an example for conflicting situations as thorough therapy would be time-consuming. Thus, antibiotics were sometimes considered to be prompt solutions to avoid tooth extraction. Three dentists claimed to be aware that they were excessing normal treatment by prescribing antibiotics, when preliminary surgical interventions would have been adequate:

*“Let’s say, an endodontically-treated molar hurts and the patient is leaving for a holiday tomorrow. And there is no chance to observe the situation or to refer the patient. I would say: ‘Okay. Before your face swells up abroad, I will write you a prescription for an antibiotic. Or you will take the antibiotic, when you notice swelling.’ […] Rarely, … but I have done this before. And, I think, the decision is hard. But it has happened.” (female dentist, 56 years, urban)*

*“And there are those cases, where I would say ‘Let’s try it this way.’—crowned tooth, metal pin, showing apical radiolucency, patient wants to leave for his holiday. ‘Do something, doctor, but don’t pull the tooth.’—‘Okay’ I would say, ‘We’ll use a sledgehammer to crack a nut’ and will take it as a makeshift.” (male dentist, 51 years, urban)*

Some dentists already described possible solutions: they asked the patients to call them the next day or recommended the consultation of a colleague or the emergency service. Furthermore, a concept of delayed prescribing was employed: some dentists asked the patients to have the prescription filled only under certain circumstances—for example continued pain or fever.

### 2.2. Patients’ Concept of Dental Illness and the Role of Dentistry

In our interviews, dentists did not report explicit patient pressure to prescribe antibiotics. On the contrary: many patients would reject the idea to take antibiotics because of bad experiences or the fear of side effects. A lot of patients, though, would express a need for fast and easy therapy as well as pain relief. At times, patients would mistake the effect of antibiotics with the effect of analgesics. One dentist described that, sporadically, patients would take a secondary gain from antibiotic therapy as being accepted as a truly ill person. Dentists claimed to not fulfil patients’ requests but to rather explain the lacking necessity of an antibiotic.

*“Sometimes people are irritated because it’s not that quick. Often, they mistake the effect of an antibiotic compared to pain medication. Because it’s common practice to take pain medication to just eliminate everything they dislike.” (female dentist, 27 years, urban)*

On the other hand, the necessity of oral health and the role of dentistry were described to be underestimated. Some dentists claimed to encounter a loss of competence when not fulfilling patients’ demands. Moreover, they felt like the patients perceived them rather as a service provider than as a physician. This, in cases, led to a semblance of resignation.

*“Well, if a patient really wants an antibiotic he’s going to get it somewhere. And if I’m the one who explains that we should watch and wait—of course, I’m the stupid one. Because there’s going to be someone else who says ‘Sure, no problem, here’s your prescription.’ […] There are a lot of areas where the one who keeps calm and explains is the one being perceived as less educated.” (male dentist, provincial)*

### 2.3. Antibiotic Prophylaxis and Conflicting Interprofessional Communication

Dentists underlined the challenges of patients with underlying medical conditions, especially heart disease as they might require prophylaxis of infectious endocarditis. It was frequently mentioned as a reason for both prescribing antibiotics and consulting fellow doctors, general practitioners (GPs) or cardiologists.

The interviewees described not feeling empowered enough to decide on the eligibility for endocarditis prophylaxis, as they did not have an equal medical knowledge on heart diseases. Despite being aware of changes in guidelines, dentists were sometimes struggling in their daily routines and thorough knowledge was vague. Therefore, soft decision factors as, e.g., patients’ experience with antibiotics or considerations of avoiding polypharmacy were sometimes applied.

*“I would never decide this by myself. And if the GP says, that he doesn’t know, I have to ask the cardiologist, what he thinks—someone has to decide, but I, as a dentist, don’t feel qualified on this matter.” (female dentist, provincial)*

To approach their uncertainties, dentists reported to regularly contact GPs or cardiologists. From time to time, this communication was not very satisfying. For example, dentists felt put down in their competency both as dentists and as fellow physicians. In line with this, they would appreciate an improved communication with GPs and cardiologists.

*“The GP is sometimes astonished that a dentist checks why a therapy should be conducted. Then one is, how can I put it, a minor-league-physician—and that’s not how I see it. Because I think, if one is concerned about the patients and cares about their well-being, you should communicate with each other. And it’s not like that anymore today, that dentistry is all about pulling and filling teeth, dentistry is much more. And I often see the patient more frequently than the GP, and at this instant it’s hard to convey this.” (male dentist, 41 years, rural)*

### 2.4. Fear of Medico-Legal Consequences

Strongly connected to the treatment of patients with heart diseases, some comments by the interviewees illustrated an underlying system of fear towards malpractice and medico-legal consequences. Insecurities about decision-making as described above were mixed with the fear of treatment failure. In case of doubt, most dentists would therefore remain pro antibiotics and avoid taking personal risks—not only in prophylactic, but also in therapeutic treatments.

*“I have to say: having to choose I would rather go with an antibiotic. If I have to decide I would always prescribe because that is just too risky. If the patients gets hospitalized afterwards everyone is going to ask: ‘Who is your dentist?’” (female dentist, provincial)*

*“I think, fear of liability is a major problem. Afterwards, if it falls back on you, everything depends on your records ‘Have you prescribed an antibiotic or not […]’. A lot of actions happen out of legal safeguarding and not because of a medical indication. Because one clever expert somewhere later might say: ‘Well, you should have done this before.’” (male dentist, provincial)*

## 3. Discussion

The quintessence was summed up by one dentist: *“But a lot of times it’s just like this: we use antibiotics because our experience shows that... Not, that it has proven to be the right choice, but at least it doesn‘t do any harm. And to make the patient and the dentist feel on the safe side, a lot of times we just prescribe it.” (male dentist, 29 years, urban)*. In our qualitative approach, we brought to light underlying explanations for non-rational antibiotic prescription behavior in German primary dental care.

The qualitative data showed that, in certain situations, dentists are conflicted by the discordance of available treatment time (e.g., the consultation in emergency services or before a holiday) and the necessity of thorough therapy. Dentists claim to be uneasy with the lacking opportunity of follow-up. Hence, they prescribe antibiotics for internal safety measurements. In addition, at times patients suffering from heart disease go beyond dentists’ medical competency. The felt lack of empowerment to make therapeutic decisions interferes with the prophylaxis of infectious endocarditis. Improvement of the conflicting communication with fellow physicians is thus wished for. In consequence of the insecurities, dentists feel pressured by possible medico-legal liability. Therefore, they tend to excess operative interventions to cover themselves legally. Dentists described moreover, to be put on a strain by patients demanding quick and easy pain relief. This collided with a feeling of deficient appreciation as a physician.

### 3.1. Comparison with Existing Literature

While questionnaire and register data on antibiotic prescribing in dentistry have been obtained over the last two decades, high-standard qualitative research has been brought forward only recently [31,32,33,34]. Most available data up to our investigation derived as secondary objective from feedback and audit or from educational interventions [18,19,20,24,26,36]. Furthermore, Stein et al. summed up possible confounders to antibiotic prescribing as part of a scoping review in 2018 [35]. The results underline our qualitative findings.

Lack of time, at times associated with patients’ demands for quick pain relief, as a trigger for excessive antibiotic treatment seems to be an almost universal phenomenon in primary dental care. Some persistent fear of health deterioration fosters over-prescribing. Cope et al., for instance, described dentists encountering time pressure and thus being more likely to prescribe an antibiotic [33]. Newlands et al. state that appropriate communication with patients about local measures was challenging due to lack of time, antibiotics then were the quick fix. Moreover, fear of loss of patients’ confidence and safety measurements were noted [34]. Palmer et al. showed that, in emergency appointments, dentists felt a need to allocate more time to patients allowing “*an active treatment rather than passive prescription*” and thus changes in practice management were considered to be required. Furthermore, patients’ expectations here were frequently causing inappropriate prescribing. Over-caution towards medically compromised patients and in cases of unclear diagnoses was described [6,7,24,37,38]. An ethnographic study stated, in line with our results, that dentists fear the risk of spreading dental infections [31]. Chate et al. found the largest significant post-audit prescription reductions for ‘patient expectation’, ‘pressure of time and workload’ and ‘uncertainty in the diagnosis’ [18]. Chopra et al. again stated that time pressure and patient expectations were the main barriers to correct treatment [20].

In previous investigations, fulfilling patient expectations in order to sustain a good patient–doctor relationship was of relevance. This sides with our finding that dentists identify as service providers. In their pilot study, Oliveira et al. claimed that there was an underlying practice to avoid clinical complications and losing patients, calling it a “defensive practice” [39]. Teoh et al. described that dentists would give distressed patients antibiotics depending on the severity of symptoms (not clinical signs) which would make the patients feel well cared for [32]. Thompson et al. have highlighted that dental practices are generally run as private businesses and are therefore reliant on nurturing relationships. In their data, dentists also described the conflict of financial burdens for patients resulting from definitive treatment [31]. In contrast to other findings, affordability of definitive treatment was not a concern of the dentists interviewed in our study [35,40]. Presumably, this is due to the coverage of basic oral health care by all German statuary health insurances.

Aspects of interprofessional communication and legal liability show to be rather scarce in previous findings. Yet, in line with our results, Soheilipour et al. cited a participant who suggested that “*antibiotic cover for patients with cardiac conditions was based partly on defensive medico-legal principles rather than on science.*” [41]. A questionnaire described that up to 48% felt a need to consult with the patients’ cardiologist for infectious endocarditis and even more wished for a specialized physician to make the decision. The authors here concluded that dentists experience difficulties in assessing risks. They therefore feel medico-legal concerns [42].

A recent review was able to define potentially modifiable determinants of behavior on antibiotic prescribing in acutely ill adults that seem to be a universal hurdle in primary care. In addition to that, they found factors that were specific to dentistry—namely, ‘procedure possible’ and ‘treatment skills’ [30]. In an ethnographic study, Thompson et al. were further able to identify never reported aspects, namely awareness of potential adverse outcomes from antibiotic use, beliefs abouts risks of antibiotics and the importance of responsible use and the accountability for dentists’ patterns of prescribing [31]. This might indicate that a certain shift might already be taking place in dentists’ attitudes towards antibiotics.

### 3.2. Strengths and Weaknesses of the Study

Although our results are supported by other findings, there are limitations by the lack of generalizability. However, this was not the aim of the investigation. The strength of qualitative methods lies in the development of hypotheses on topics that have not been explored before. Taking this into account, we ensured rich insight into our participants’ perspectives. Different levels of triangulation were used in order to increase validity, e.g., interviews were supported by subsequent focus group discussions and both, data collection and analysis, were undertaken by a multidisciplinary team [43]. Even though the data approximated saturation, including GPs or cardiologists might have further underlined certain aspects on cooperation. Possibly due to differences in socioeconomic upbringing, there still remain significant differences of antibiotic consumption between eastern and western German regions (with former West Germany prescribing more) [44]. Results may thus not be fit for extrapolation to all regions in Germany. Additionally, our findings may add to understanding inappropriate prescribing in what is predominantly understood as the Western world—as different as those health care systems may be, they will most certainly not reflect on needs and barriers in low- and middle-income countries.

### 3.3. Implications

As different levels of struggle with antibiotic prescribing were discovered, several strategies to cope with this problem were indicated in our findings and the comparison to similar literature.

Firstly, empowering dentists to adhere to their own medical principles—and communicating this with their patients—might be of great use. Having evidence that patients frequently neither hope for nor expect an antibiotic supports this idea. As Anderson showed, most emergency patients seek for pain relief and a greater certainty regarding the cause of their problem. A desire or expectation for antibiotics was rare [45]. Dentists could be encouraged to focus on pain relief and reassurance through optimized communication skills. Research on acute respiratory infections in general practice showed that good doctor–patient communication can significantly lower antibiotic prescription rates [46,47]. Yet, none of the investigations on dental antibiotic prescriptions so far focused on shared decision making.

Moreover, in our evaluation dentists experienced their education on antibiotics as insufficient and predominantly arranged by pharmaceutical firms. We found that the interviewees underestimate their own prescription figures and adhere to their personal empiricism in therapeutic decision processes. Cope et al. further showed that dentists believe their contribution to antibiotic resistance to be far less than medical colleagues [36]. The access to current guidance in Germany was especially compromised as the most recent guideline was issued in 2002 until in 2016 an updated version was published by the German Association for Dental, Oral and Maxillofacial Medicine (DGZMK). Nevertheless, even then it stated that operative intervention should be central in the treatment of abscessing infections [48,49]. This coheres with international recommendations [19,50]. An Australian interview study described reasons for the choice of an antibiotics to be what they were taught in university, advice from colleagues and if patients were familiar with it. Moreover, pack sizes would determine the duration of a treatment [32]. A recent UK-study found antibiotic prescribing probabilities associated with lack of postgraduate qualification and having a dental qualification from a non-UK university [51]. Not only publishing, but promoting up-to-date therapeutic recommendations and implementing concepts to pre- and post-graduate education hence might rationalize antibiotic prescribing.

In addition, our study suggests that the fear of medico-legal consequences and the conflicting interprofessional cooperation might be of even greater significance compared to pre-available data and also in contrast to primary care. Fear of criticism online and medico-legal considerations were also found by Teoh et al. Educating patients on the unnecessary use of antibiotics was avoided in order to maintain good relationships. [32] Recognizing that the risk of adverse events can exceed the benefits of antibiotic use in certain patients should be encouraged [52].

Prescription feedback furthermore could help dentists reflect upon their level of antibiotic issuing. Cope et al. describe that dentists wished for greater incentives to firstly provide operative treatment [33]. In line with this, one of our interviewees claimed: *“It might be utopic within our compensation system, but really physicians should be rewarded for accompanying someone their whole life without getting diseases.” (male dentist, provincial)*.

## 4. Materials and Methods

In order to disclose underlying behavioral mechanisms, qualitative methods are often employed. In contrast to common quantitative methods used in health care research, objective measurements are not the goal of qualitative approaches. This methodology is especially useful when no presumptions are made and limited or no prior theory exists. It may generate hypotheses and thus provide new perspectives to health care [53,54,55]. Data derived from qualitative studies can, e.g., be beneficial in preclinical phases of cluster randomized studies as these approaches provide hypotheses to be tested in process [56].

In this study we followed a two-step approach: we performed narrative interviews. The emerged concepts and solutions were afterwards further reflected in focus group discussions.

The investigation was set in Mecklenburg-Western Pomerania, a predominantly semirural region of north-eastern Germany. Most dentists worked in or within the proximity of a larger town.

### 4.1. Narrative Interviews

Two researchers, a physician and a sociologist trained in qualitative methods, conducted open-ended in-depth interviews with nine dentists in primary care. An interview guideline with narrative stimuli was previously developed within the interprofessional team (Table 2). Hence, instead of using a fixed catalogue of questions dentists were given the opportunity to reflect upon their own experiences.

A purposeful sampling strategy was employed to reflect the range of diverse dental practitioners. Recruitment was performed via invitational letters to dentists within a 50 km range of Rostock, Germany and followed up by telephone. The dentists had been previously selected based on known socio-demographic characteristics. Eight dentists worked as GDPs, one had undergone further training and worked as maxillofacial surgeon. Interviews were conducted at the dentists’ practices or on campus of the Rostock University Medical Centre. All interviews were on a one-to-one-level. Interviews lasted on average 33 min (17–57 min).

Recruitment was stopped after nine interviews as theoretic data saturation was approximated, meaning no further or new theoretical insights were gained on which the categories could have been further developed [54]. At this point the researchers judged to have a rich comprehension of dentists’ perspectives and decided to complement and triangulate hypotheses by conducting focus group discussions.

### 4.2. Focus Group Discussions

Two focus group discussions were arranged. Recruitment was performed via randomized invitational letters to practice based primary care dentists within a 50 km range of Rostock, Germany and followed up by telephone. The two consecutive focus group discussions were attended by four respectively five dentists and were on average 86 min long. They were set at the Rostock Institute of General Practice and lead by an experienced female dentist and a male general practitioner highly trained in health care research.

Theories from the interviews were reflected and case reports discussed. Furthermore, possible intervention approaches and materials we had developed based on our previously gained data were evaluated for approval and feasibility. This shall be described elsewhere.

### 4.3. Analysis

Both, interviews and focus group discussions were audio-recorded and transcribed verbatim. In order to maintain confidentiality, details on dentists were removed and names were pseudonymized. In the process of data collection and analysis field notes were taken and memoing was employed [53].

Data collection and analysis took place concurrently. Interviews were analyzed by two researchers (one sociologist, one physician), using a combination of deductive and inductive thematic analysis. The initial coding strategy was developed using an inductive approach: the structure of analysis derived from data as no predetermined theory was specified in advance. Subsequent interviews were coded separately and emerging themes were identified. Consensus was reached through discussion. The analysis was conducted parallel to interviews, while the focus group discussions were evaluated last to support or neglect pre-established hypotheses. Continuous exchange within the multidisciplinary team of dentists, sociologists, health scientists and general practitioners enriched comprehension and validity.

To facilitate data-coding, we employed NVivo 9© (QSR International), a software for qualitative data analysis.

## 5. Conclusions

Our analysis for the first time aimed at deeply understanding reasons for irrational prescribing in German primary dental care and discovering specific barriers. Our hypotheses concord with preliminary data, mainly from the UK, but highlighted medico-legal concerns and interprofessional communication as even greater barriers than described before. Our findings implicate that the empowerment of dentists concerning their own insecurities might improve antibiotic prescribing. Further investigations on well-targeted interventional concepts built upon our and other findings thus need to be fostered, as dentistry shows a huge potential for improvement considering the percentage of prescriptions overall and the rates of estimated inappropriate antibiotic use. Using adapted, yet common, interventional concepts derived from primary care may help this cause and help define to which extent interventional concepts can be transferred and adapted from one medical field to another.

## Figures and Tables

**Table 1:** Sample description.

		Interviews	Focus Group Discussions
Sex	Female	4	3
	Male	5	6
Age range (years)		27–56	/
Level of urbanisation	Rural (up to 5000 inhabitants)	3	2
	Provincial (5000–30,000 inhabitants)	0	5
	Urban (more than 30,000 inhabitants)	6	2
Specialisation	General dental practitioner (GDP)	7	7
	Maxillofacial surgeon	1	0
	GDP with focus on implantology and/or periodontology	1	2
Total		9	9

**Table 2:** Interview guide.

I am interested in learning about the use of antibiotics in the dentistry. Can you recall a situation where you used antibiotics? Please tell me about it. (Alternatively, please recall a situation in which you used antibiotics on a patient and tell me about it). You can take as much time as you like. I will not interrupt you for now, just take notes and come back to it later.
Suggested external follow-up questions: - Can you think of situations when you had a hard time deciding whether or not to use antibiotic therapy? (Alternatively, can you think of examples of positive or negative experiences with antibiotic use)? - Can you recall a situation in which your proposed therapy and the patient’s wishes and expectations differed? (Alternatively, what role do patient wishes and expectations play for you in antibiotic prescribing)? - For what symptoms do you generally prescribe antibiotics? - What did you learn about prescribing antibiotics in your professional training or continuing education? What did you experience in the process?

## Data Availability

The data analyzed during the current study are available from the corresponding author on reasonable request.

## Abbreviations

GDP—general dental practitioner; GP—general practitioner.
